# Verification of the utility of the social responsiveness scale for adults in non-clinical and clinical adult populations in Japan

**DOI:** 10.1186/s12888-014-0302-z

**Published:** 2014-11-18

**Authors:** Reiko Takei, Junko Matsuo, Hidetoshi Takahashi, Tokio Uchiyama, Hiroshi Kunugi, Yoko Kamio

**Affiliations:** Department of Child and Adolescent Mental Health, National Institute of Mental Health, National Center of Neurology and Psychiatry, 4-1-1 Ogawa-Higashi, Kodaira, Tokyo 187-8553 Japan; Department of Mental Disorder Research, National Center of Neurology and Psychiatry, Tokyo, Japan; Department of Faculty of Human Development, Fukushima University Graduate School, Fukushima, Japan

**Keywords:** Autism spectrum disorder, Adult, Screening, Questionnaire, Psychiatric population

## Abstract

**Background:**

Recently great attention has been paid to the still unmet clinical needs of most adults with autism spectrum disorder (ASD) who live in the community, an increasing number of whom visit psychiatric clinics to seek accurate diagnosis and treatment of concurrent psychiatric symptoms. However, different from the case of children diagnosed with ASD in childhood, it is difficult in adults to identify the ASD symptoms underlying psychopathology and to differentiate ASD from other psychiatric disorders in general psychiatric practice. This study aimed to verify the utility of the Social Responsiveness Scale-Adult version (SRS-A), a quantitative measure for identifying ASD symptoms, in non-clinical and clinical adult populations in Japan.

**Methods:**

The total sample aged 19 to 59 years consisted of a non-clinical population (*n* =592) and clinical population with and without ASD (*n* =142). We examined score distributions of the Japanese version of the scale, and the effects of gender, age, and rater on the distribution. We analyzed factor structure and internal consistency in the non-clinical normative sample, and analyzed convergent, divergent, and discriminative validities in the clinical sample. We applied receiver operator characteristic (ROC) analysis to determine optimal cutoff scores discriminating the ASD clinical population from the non-ASD clinical population.

**Results:**

The score distributed continuously, which replicated findings in children. For non-clinical adults, except in men aged 19 to 24 years, we found no or few gender, age, or rater effects. Both single- and two-factor models were supported for adults. Total SRS-A scores demonstrated high internal consistency and capably discriminated adults with ASD from those with non-ASD psychiatric disorders such as major depressive disorder, schizophrenia, and bipolar disorder with an overlap across diagnoses. Moderate to high correlations of the SRS-A with other-rated ASD measures indicated sufficient convergent validity. Based on the ROC analysis, we recommend cutoff points by gender for use in clinical settings.

**Conclusion:**

This study provides additional supportive evidence that the Japanese version SRS-A can reliably and validly measure ASD symptoms in non-clinical and clinical adult populations, and thus can serve as a useful tool for ASD research as well as for secondary screening in Japanese adults.

## Background

According to a recent epidemiological study [1], autistic spectrum disorder (ASD) is currently estimated to be 1% of the adult population, a figure that approximates that in the child population [2]. Recently, ASD in adulthood has attracted considerable interest in the field of general psychiatry. It has been identified that most adults with ASD living in the community still had unmet clinical needs and are socially disadvantaged [1,3]. In line with this worldwide trend, in Japan, an increasing number of adults with ASD visit psychiatric clinics with a diverse range of chief complaints, seeking either accurate diagnosis and a medical certificate needed to receive transition support for employment or treatment of concurrent psychiatric symptoms such as depression or anxiety [4]. However, unlike in children diagnosed with ASD, clinical manifestations in adult patients first diagnosed with ASD in adulthood are often complex: deficits in social reciprocity tend to be less apparent in adults with high-functioning ASD, especially outside situations that demand responses to complex social cues, or when adults with ASD mask their deficits using compensation strategies. For these reasons, it is difficult to identify ASD symptoms underlying adulthood-onset psychopathology and differentiate ASD from other psychiatric disorders in general psychiatric practice, which can lead to misdiagnosing ASD symptoms as psychosis [5].

In the Diagnostic and Statistical Manual of Mental Disorders, Fifth Edition (DSM-5) [6], a category of pervasive developmental disorders in the Diagnostic and Statistical Manual of Mental Disorders, Fourth Edition, Text Revision (DSM-IV-TR) [7] has been converted to a new category of ASD in which ASD severity is quantitatively rated according to current difficulties. Further, because of a nonexistent natural boundary between affected and unaffected individuals [8,9], the DSM-5 has a new category of social (pragmatic) communication disorder for individuals with marked deficits in social communication but who do not otherwise meet ASD criteria (i.e., those with subthreshold ASD) [6]. The availability of quick, easy-to-use, and validated screening tools for identifying autistic traits and symptoms for psychiatric patients would help make appropriate diagnoses, reduce misdiagnoses, and plan appropriate treatment or support according to individual patient needs.

To date, the few self-report questionnaires available for identifying ASD in adulthood include the autism-spectrum quotient (AQ) [10-12], the Ritvo Autism Asperger’s Diagnostic Scale [13], or the Ritvo Autism Asperger Diagnostic Scale-Revised [14]. By contrast, a few questionnaires such as the Social Responsiveness Scale-Adult version (SRS-A) [15], or the Autism Spectrum Disorder in Adults Screening Questionnaire [16] must be completed by another adult (e.g., family member, close friend, or professional). The SRS-A was modified from the SRS [17], a quantitative measure of autistic traits in children. The original SRS has been extensively validated in clinical and subclinical child populations as well as in general child populations not only in the U.S. [8,15,17-19] but also in Europe [20], South America [21], and Asia [9,22]. The SRS can distinguish children with ASD from children with any other or no psychiatric disorder and is generally unrelated to IQ in the normal range [9]. The SRS, a quantitative measure of autism, is also sensitive to autistic traits and symptoms even in subthreshold ASD conditions [9]. It is extremely useful for research purposes such as genetic epidemiological research [19,23] or in research assessing brain-behavior relationships [24]. Its utility for detecting autism-related genetic loci [25] or cross-species research [26] has been suggested.

However, at this time, only a few validation studies of the SRS-A exist [15,27,28]. Therefore, the main purpose of this study was to determine the score distribution of the Japanese version SRS-A in a non-clinical Japanese adult population, and to assess its factor structure, reliability, and validity. Based on our findings in clinical populations with and without ASD, optimal cutoff scores are recommended.

## Methods

### Participants

The normative sample included 592 participants (257 university students and 335 private company or hospital workers; men, 41.6%) aged 19 to 59 years. Another adult who knew each participant well, such as a parent, spouse, sibling, or close friend, answered an SRS-A questionnaire with complete anonymity. After excluding survey responses with missing data, we used complete data sets from 458 participants (men, 45.2%) (Tables 1 and 2). Excluded responses (*n* =134) were 22.6% of the obtained responses and most often were from participants in adolescence (56.7% of incomplete responses), followed by those in middle age (26.1%) and early adulthood (17.2%). Among them (*n* =134), 97 (72.4%) did not specify who the rater was, and the rest were excluded due to missing SRS-A answers. In this study, we included only complete SRS-A questionnaires with responses having specified gender, age, and rater data for further analyses.

**Table 1:** **Mean total scores of the Social Responsiveness Scale for Adults** (**SRS**-**A**) **in the normative sample by sex and age**

	**SRS-A total score**				
**Age group (years)**		**Male**		**Female**	
	**Total** ***N***	***N***	**Mean (** ***SD*** **)**	***N***	**Mean (** ***SD*** **)**
Adolescence (19–24)	183	87	53.4 (27.8)***	96	36.5 (21.2)***
Early adulthood (25–39)	122	53	36.2 (25.1)	69	36.3 (22.6)
Middle age (40–59)	153	67	35.9 (26.1)	86	30.1 (20.0)
Total	458	207	43.3 (27.8)	251	34.3 (21.3)

***Men scored significantly higher than women in the 19–24 age band (*p* < .001).

**Table 2:** **Mean total scores of the SRS**-**A in the normative sample by rater and number of participants by rater**, **gender**, **and age band**

**Rater type**	**Mean** **(** ***SD*** **)**	***N*** **(** **M** **;** **F** **)**	**Adolescence** ***N*** **(** **M** **;** **F** **)**	**Early adulthood** ***N*** **(** **M** **;** **F** **)**	**Middle age** ***N*** **(** **M** **;** **F** **)**
Mother	39.8 (25.2)	148 (49;99)	126 (44;82)	21 (4;17)	1 (1;0)
Father	57.9 (26.1)	49 (40;9)	47 (39;8)	2 (1;1)	0 (0;0)
Spouse	33.1 (21.7)	205 (98;107)	1 (0;1)	79 (40;39)	125 (58;67)
Siblings, friends, or others	34.5 (20.0)	56 (20;36)	9 (4;5)	20 (8;12)	27 (8;19)
Total	38.2 (24.7)	458 (207;251)	183 (87;96)	122 (53;69)	153 (67;86)

The validation sample consisted of 65 patients diagnosed with ASD (ASD group; men, 67.7%) and 78 patients diagnosed with non-ASD psychiatric disorders (non-ASD group; men, 50%) (Table 3). Both the ASD and non-ASD clinical groups included research volunteers registered at the National Center of Neurology and Psychiatry (NCNP) and patients from several specialized developmental clinics. Our research team that included specialized child psychiatrists diagnosed participants in the ASD group according to DSM-IV-TR (20: autistic disorder; 28: Asperger’s disorder; 17: pervasive developmental disorder-not otherwise specified [PDD-NOS]). In addition to clinical diagnosis, we evaluated 51 of the 65 participants using either the Autism Diagnostic Observation Schedule (ADOS) or a semi-structured interview scale developed, validated, and widely used in Japan [23]. Participants in the non-ASD group were diagnosed with any DSM-IV-TR Axis I mental disorder (30: major depressive disorder; 26: schizophrenia including schizoaffective disorder; 17: bipolar I and II disorders; 4: anxiety disorders; 1 personality disorder) based on either a brief standardized interview (the Mini-International Neuropsychiatric Interview) or clinical assessment by a psychiatrist. All participants were clinically judged to have intellectual functioning within the normal range. The intelligence quotients (IQs) of 29 participants in the ASD group and 15 participants in the non-ASD group were confirmed by formal cognitive testing (mean IQ, 104.4 ± 13.8, 91.6 ± 12.2, respectively). All participants in the ASD group were rated by their mothers, while those in the non-ASD group were rated by their mothers or spouses.

**Table 3:** **Mean total scores of the SRS**-**A of the ASD and Non**-**ASD Groups**

	**ASD group**	**Non-ASD group**
	**mean (** ***SD*** **), range**	**mean (** ***SD*** **), range**
*N* (Male: Female)	65 (44:21)	78 (38:40)
Age Mean (SD), Range	27.3 (7.7),19-51***	34.8 (10.6) , 20–59
SRS-A scores	87.6 (29.1), 32-153***	54.7 (24.4), 12-106
Rater Mother	65 (44:21)	46 (24:22)
Spouse	0	32
*Mother ratings*		
Age	26.3 (6.4), 19-51^✝^	28.4 (6.5), 20-43
SRS-A scores Male	89.4 (29.1), 33-167^***^	64.3 (34.8), 12-106
Female	79.8 (26.9), 42-119^**^	57.2 (25.7), 13-106
Total	86.9 (28.7), 33–167^***^	60.9 (30.6) , 12–106

**p < .01; ***p < .001; ^✝^ *p* > .05.

### Measures

#### The social responsiveness scale for adults

The Social Responsiveness Scale for Adults (SRS-A) is a 65-item questionnaire of autistic traits used with adults, with modified wording of the original SRS [17]. Similar to the SRS for children, each SRS-A item is scored on a 4-point scale with total scores ranging from 0 to 195, with higher scores indicating higher degrees of social impairment. For the Japanese adaptation, the original SRS-A was translated into Japanese by members of our research team (Y.K., H.T.) with permission from Western Psychological Services (WPS). In translating the SRS-A into Japanese, translations were adopted from the Japanese version of the SRS [8] whenever possible to ensure consistency across the child and adult versions. This translation was back-translated into English by independent translators and the last author (Y.K.), and one of the developers (J.C.) confirmed item equivalence in the two languages. The original developers and WPS then approved the final Japanese version, which we used in this study.

#### The autism diagnostic observational schedule

The Autism Diagnostic Observational Schedule (ADOS) is a semi-structured behavioral assessment of social interaction, communication, and stereotyped behaviors. The original diagnostic algorithm generates scores for each of three domains of autism. Diagnostic classification is made by exceeding two cutoffs: autism and autism spectrum. To meet the ADOS criteria for autism or autism spectrum, the cutoff must be reached in both the social and communication domains and the sum of social and communication scores. In this study, we used the sum scores of the Japanese version ADOS (Module 4) [29] to assess participants in the ASD group.

#### The pervasive developmental disorders-autism society Japan rating scale

The Pervasive Developmental Disorders-Autism Society Japan Rating Scale (PARS) is a semi-structured interview useful for children and adults, and its scores are correlated with the scores of the Autism Diagnostic Interview-Revised, demonstrating criterion-related validity of the PARS [30]. In this study, to assess participants in the ASD group, we used the PARS version for adolescents and adults, whose reliability and validity were demonstrated [31] and whose scores were strongly correlated with the SRS scores for adolescents (*r* =0.77, *p* < .001) [32].

#### The autism-spectrum quotient-Japanese version

The AQ is a 50-item self-report scale for identifying high-functioning autism in individuals with normal intelligence [10,12]. Each item is scored on a 4-point scale with total scores ranging from 0 to 50; higher scores indicate more severe autism. In this study, we used the Japanese version of the AQ (AQ-J) [11] to assess autistic traits of participants of both ASD and non-ASD groups.

### Analysis

In our normative data collection, the gender ratio in each age band was not significantly different (*χ*^2^ = 0.68, *ns*) (see Table 1). However, there was a natural selection bias for rater type depending on the participant’s gender (*χ*^2^ = 37.6, *p* < .001) or age (*χ*^2^ = 346.7, *p* < .001) (see Table 2). Therefore, instead of performing an analysis of variance (ANOVA) using gender, age band, and rater type as between-subjects factors for this sample, a two-way ANOVA was performed to reveal the effects of gender and age (two factors, gender × age band; adolescence, 19–24 years; early adulthood, 25–39 years; and middle age, 40–59 years) with total SRS-A scores of the normative sample as a dependent variable. Second, in order to examine rater-dependent effects in the normative sample, we conducted a two-way ANOVA for adolescent participants with total SRS-A scores as a dependent variable, and rater type (mother, father) and gender (male, female) as between-subjects independent variables, because a substantial number of adolescents were rated by either mothers or fathers. Third, we performed confirmatory factor analysis (CFA) to examine the most parsimonious model suggested by extensive prior research on the SRS [9,18,20,33]. To do so, we used MPlus 7.11 with a robust weighted least squares estimator on the normative sample and treated the SRS-A data as ordered categorical variables. Fourth, we calculated internal consistency (Cronbach’s α) for 65 total items in the normative sample. Fifth, to examine convergent and divergent validities, we computed Pearson’s coefficients between the SRS-A, ADOS, PARS, AQ-J, and IQ scores for the validation sample. To consider how well the SRS-A distinguishes between ASD and non-ASD psychiatric disorders, we performed *t*-tests, one-way ANOVA, and receiver operating characteristic (ROC) analyses for the validation sample. We compared mean SRS-A total scores between the ASD and non-ASD groups using a *t*-test, and between diagnostic subcategories within each group using one-way ANOVA. Based on ROC, we determined optimal cutoff points for ASD screening. All analysis except for CFA was performed using SPSS 17.0 J for Windows. We used an alpha level of .05 for all statistical tests.

### Ethical considerations

The study protocol was approved by the Ethics Committee of the NCNP, Japan. For the validation sample, we obtained written informed consent to participate in this study from adult participants and the caregivers of each child.

## Results

### SRS-A total scores of the normative sample and effects of gender, age, and rater

**Figure 1:**
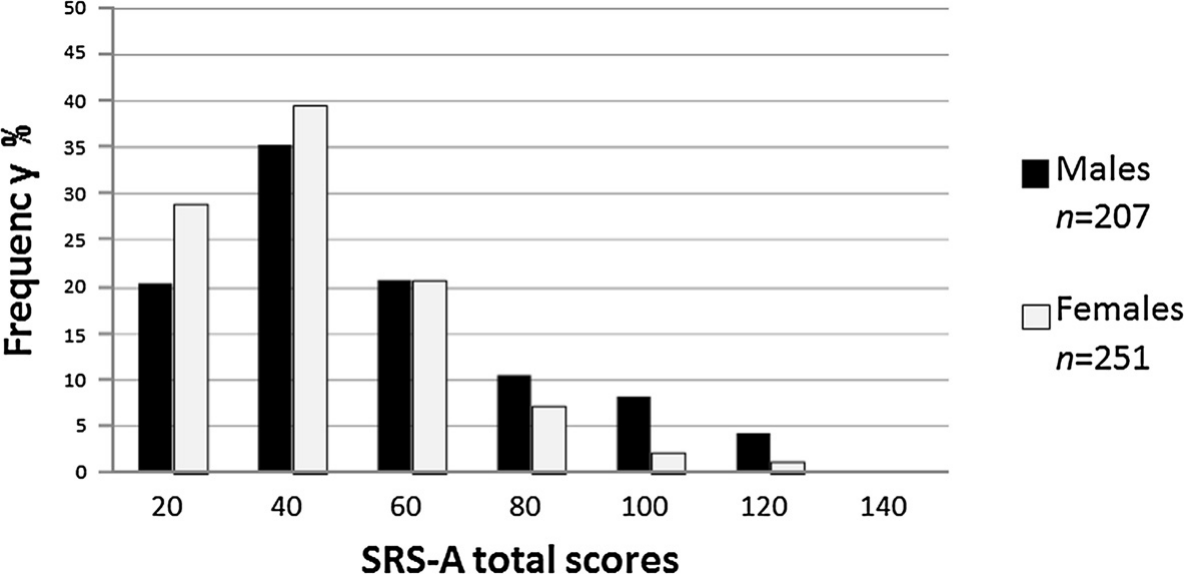
**Distribution of Social Responsiveness Scale for Adults** **(** **SRS** **-** **A** **)** **total scores in the normative sample** **(** ***n*** **=** **458** **).**

In the normative sample, the distribution of SRS-A total scores for each gender showed that men generated higher scores than women (Figure 1), as in the SRS for children. Table 1 shows mean (*SD*) SRS-A scores by gender and age. The main effects, gender (*p* = .003, *η*^*2*^ = 0.02) and age (*p* < .001, *η*^*2*^ = 0.05), and the interaction between gender and age (*p* < .005, *η*^*2*^ = 0.02) were all significant, but with a small effect size. As for simple main effects, scores were not significantly affected by different age groups in women, whereas adolescent men scored significantly higher than men in early adulthood and middle age, with a moderate effect size (*ps* < .001, each with *d* =0.64, 0.64). We observed a significant gender difference only between adolescent men and women (*p* < .001, *d* =0.68).

Regarding the rater, our sample had a natural selection bias for rater type depending on the participant’s gender or age (see Table 2). Although only two participants in early adulthood were rated by fathers and only one in middle age were rated by parents, 95% of adolescents were rated by either a mother or father. Mothers of adolescents rated their daughters (82/126) twice as often as they did their sons (44/126) in this sample. In contrast, fathers of adolescents rated their sons (39/47) five times as often as their daughters (8/47). The majority of participants in early adulthood (65%) and middle age (82%) were rated by their spouses. The ANOVA results for adolescent participants revealed no significant interaction between gender and rater type, but did show a significant main effect of rater type (*p* = .01, *η*^*2*^ = 0.03) and gender (*p* = .001, *η*^*2*^ = 0.06). That is, father ratings (64.0 ± 28.5, 41.2 ± 18.3, for men and women, respectively) were significantly higher than mother ratings (46.1 ± 24.8, 35.6 ± 22.8, for men and women, respectively) for each gender in this age band. However, because this study was not designed to systematically examine the rater effect, we are unable to draw a conclusion about rater-dependent effects on scores according to the participant’s age or gender from this sample.

### Factor structure

The single factor model was subjected to CFA using all 65 items from the normative sample. The estimate for root mean square error of approximation (RMSEA) was 0.048 and the 90% confidence interval (CI) was 0.045–0.050. An acceptable model should have an RMSEA less than 0.05, and the probability that the RMSEA of the single factor model is less than 0.05 is 97.2%, indicating a good model fit. In addition, the comparative fit index (CFI) and Tucker Lewis Index (TLI) were 0.894 and 0.890, respectively, where these values close to 0.90 indicate a reasonable fit. These findings provide further support for a single factor model underlying the multiple aspects of autistic traits and symptoms. Given that Frazier et al. [33] validated the two-factor model of ASD proposed by DSM-5 [6] based on data from a large sample of children and adults, we tested whether the two-factor model (one factor comprising 53 social-communication [SC] items and another comprising 12 autism mannerisms [AM] items) has a good fit. We found that the two-single factor model adequately fits, to almost the same degree as the single factor model (RMSEA, 0.047; 90% CI, 0.045–0.049; probability of RMSEA <0.05, 98.9%; CFI, 0.896; and TLI, 0.893). Very high correlations were observed between SC and AM (*r* =0.91, 95% CI: 0.890–0.935). The high correlation between these two ASD domains suggests that total scores will be adequately represented by a single factor structure.

### Reliability

Cronbach’s α for the normative sample was 0.96, indicating strong internal consistency.

### Validity

#### Convergent validity

The correlation between the SRS-A and PARS scores was relatively strong (*n* =14, 12 ASD, 2 non-ASD, *r* =0.62, *p* = .019). The correlation between the SRS-A and the ADOS module 4 scores was moderate (37 ASD, *r* =0.34, *p* = .037). The correlation between the SRS-A and AQ-J scores (*n* =76, men 52.6%; 33 ASD, 43 non-ASD; mean age ± SD [range], 35.5 ± 11.4 [20–59] years) was significant but weak (*r* =0.25, *p* = .030).

#### Divergent validity

For the available IQ data (*n* =44), the SRS-A score did not significantly correlate with IQ (*r* = −0.09, *ns*).

#### Discriminative validity

The ASD group scored significantly higher than the non-ASD group (*p* < .001, *d* =1.07) (Table 3). When SRS-A scores were compared between the groups according to gender, both men (*p* < .001, *d* =1.14) and women (*p* = .005, *d* =0.84) scored significantly higher in the ASD group than in the non-ASD group. Further, gender differences in SRS-A scores were not significant in either the ASD or non-ASD group. Because the findings from our normative sample scores suggested rater bias, only mother ratings were compared between groups (Table 3). Again, participants with ASD scored significantly higher than those without ASD (*p* < .001, *d* =0.88). Age of this subgroup did not significantly differ between groups. Within groups, SRS-A scores revealed no significant gender differences. SRS-A scores in the ASD group did not significantly differ by subcategory (autistic disorder, 99.2 ± 28.3; Asperger’s disorder, 83.7 ± 31.2; and PDD-NOS, 80.4 ± 23.4). Within the non-ASD group, SRS-A scores did not significantly differ by co-occurring disorder (major depressive disorder, 48.9 ± 26.9; schizophrenia, 59.8 ± 25.9; bipolar disorder, 53.7 ± 23.0; other disorders, 64.8 ± 36.7).

**Figure 2:**
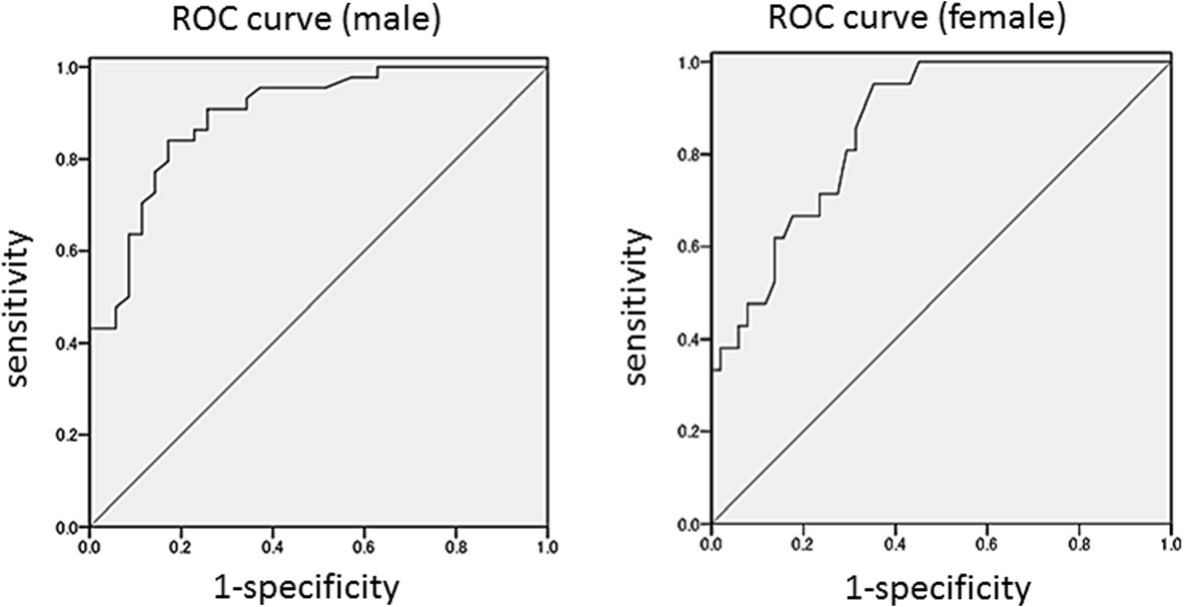
**Receiver operating characteristics** **(** **ROC** **)** **curve of the SRS** **-** **A conducted for the validation sample** **(** ***n*** **=** **143** **) (** **Left** **:** **male** **,** **Right** **:** **female** **).**

Due to the gender-biased score distributions found in the normative sample (Figure 1), we generated a ROC curve for each gender in the validation sample (Figure 2). Area under the curve was 0.896 (95% CI: 0.83–0.97, *p* < .001) for men and 0.859 (95% CI: 0.78–0.94, *p* < .001) for women, with both moderately able to discriminate ASD and non-ASD psychiatric disorders in a clinical population. Youden index values (sensitivity + specificity-1) were maximized at a score of 65 for men and 52 for women, at which sensitivity was 0.84 and specificity was 0.81 for men, and sensitivity was 0.95 and specificity was 0.61 for women. These cutoff values are highly sensitive to ASD among various psychiatric disorders and might be suitable for identifying possible ASD in clinical settings. To make a definite diagnosis, the next step is a detailed interview with appropriate examination and history taking.

## Discussion

This study provides some evidence supporting the continuous distribution of autistic traits in a non-clinical adult population using the Japanese version of the SRS-A, and the satisfactory reliability and validity of the Japanese version SRS-A for adults aged 19 to 59 years. The Japanese version SRS-A was shown to be capable of detecting ASD and autistic traits/symptoms among a psychiatric population and also screening for ASD. The finding of continuous distribution of autistic traits in a non-clinical adult population as measured by the SRS-A and its single factor structure is closely similar to what has been observed in children [9,18,20]. The SRS-A provides additional evidence about the nature of the autistic spectrum [6]. Mean SRS-A scores corresponded to mean parent-rated SRS scores in Japanese children [9]. The effects of gender or age on SRS-A scores among a non-clinical adult population in this study were overall minimal, being similar to previously reported parent-rated scores in a child population aged 7 to 15 years [9]. Only in adolescents did we observe a significant gender difference with a moderate effect size. However, this male-dominant difference found in adolescent participants could be explained by age-dependent rater bias. Regarding rater effects, we could examine these for adolescent participants only due to practical restraints of the collected data. Our results demonstrated that, father ratings were significantly higher than mother ratings for adolescents. In an examination of a twin sample, Constantino and Todd (2005) reported strong parent-offspring correlations of subthreshold autistic traits as measured by the SRS [27], indicating that autistic traits are strongly heritable for the pairing. According to that study, the father-offspring correlation was higher than that of the mother-offspring pairing, and that of the father-son pairing was the strongest at 0.58. It is unclear whether such father-son similarity in social responsiveness might have affected the extremely high father ratings of their adolescent sons in this study. Given that the special status of fathers as the rater has not been observed in the U.S. standardization sample [15], pp. 44, alternatively our finding might better be explained by Japanese fathers’ high expectations of their sons approaching adulthood (i.e., they are no longer children but also not yet independent adults). The interrelationship between rater type, gender, and age remains to be replicated by larger-scale studies in Japan and in other cultures. Taken together, when interpreting the information SRS-A provides, we must keep in mind various factors, especially rater type in terms of social expectations within sociocultural contexts.

From the viewpoint of cross-cultural comparison, the score distribution in this study is comparable to that of U.S. data [15,27]. The mean scores in our non-clinical sample rated by various raters (19–59 years; 43.3 for men, 34.3 for women) are comparable to those rated by mixed raters (18–89 years; 42.2 for men, 38.8 for women) reported in the SRS-2 Manual [15], pp. 44. As for spouse ratings of participants in early adulthood or middle age, the mean scores in our non-clinical subsamples (33.1, male 48.8%) were very similar to those of U.S. data rated by spouses (30–55 years; 31.7 for men, 30.0 for women) [27]. However, the scores in our sample were lower compared to those of German adults with typical development (19–79 years, 55.5 for mixed sex) as reported by Bölte [28]. The reason for this discrepancy between Bölte’s and our scores is not clear because rater-type details were not mentioned in Bölte [28]. As emphasized in the SRS-2 Manual [15] as well as in Bölte [28], the effect of rater type, which varies depending on an adult’s age, gender, or living situation, might be crucial and should be systematically studied in future research.

Regarding convergent validity, correlations between the SRS-A and ADOS or PARS ranged from moderate to relatively strong (the latter two of which were assessed based on direct or indirect clinician observation), and these correlations provide support that the Japanese version SRS-A measures the same clinical aspects of the autism spectrum as do validated measurements. By contrast, the weak correlation between the SRS-A and AQ-J might be because the AQ-J is self-rated, and suggests that these two questionnaires might measure different aspects of the autistic spectrum.

Although the Japanese version SRS-A capably discriminated adults with ASD from those without ASD but having any other psychiatric diagnosis such as major depressive disorder, schizophrenia, or bipolar disorder, we observed no gap but rather an overlap in the score distribution between the two clinical groups (with and without ASD) in our study. This finding is consistent with that observed for school-age children [9], although the non-ASD child clinical population in that previous study [9] included adjustment disorder, attention-deficit/hyperactivity disorder (ADHD), and anxiety and other disorders, making it more diverse than the non-ASD adult clinical population in the present study. Recent genetic, molecular, and cytologic research highlights shared contributory mechanisms between ASD and major adult-onset psychiatric disorders (i.e., major depressive disorder, bipolar disorder, schizophrenia [34], and behavioral-cognitive commonalities [5,35]). Further, concurrent depression and anxiety symptoms, which are likely accompanied by transient psychotic symptoms, are found not only in individuals with ASD but also in those with subthreshold autistic symptoms [36,37]. Given this transdiagnostic commonality, the overlap in the SRS-A score distribution in the present study suggests that a proportion of the non-ASD clinical population might have autistic traits/symptoms despite having subthreshold ASD, which can lead to clinical difficulties in differential diagnosis. Because such clinical uncertainty from concurrent psychiatric symptoms is likely to result in misdiagnosis that overlooks ASD, our result that the SRS-A has discriminative ability for ASD with high sensitivity would prove the clinical usefulness of the instrument as a secondary screening tool in psychiatric practice. From a therapeutic viewpoint, it is important to detect autistic symptoms of not only threshold ASD but also subthreshold autistic conditions among various psychiatric populations so that appropriate treatment based on a comprehensive clinical evaluation can be given [36,37]. To this end, we recommend cut-off scores of 65 for men and 52 for women, which are similar to those for Japanese children [9], even though there are some adults who do not meet the diagnostic criteria of ASD above the cut-off.

This study has several limitations. First, our sample size was small, from which we examined a subsample using validated instruments measuring autistic traits and severity or IQ. The non-ASD clinical group mainly included participants diagnosed with schizophrenia and depressive or bipolar disorders, although other various psychiatric comorbidities are also common in adults with ASD, notably anxiety disorder and ADHD [38]. Replication in a larger psychiatric population including anxiety disorder and ADHD is needed. Second, our normative sample did not include individuals aged 60 or more for whom the SRS-2 manual gives higher scores [15], pp. 44. Third, we did not examine inter-rater agreement, which can explain differences due to rater type found in this study as well as test-retest reliability. Fourth, we did not examine the self-report SRS-A. A comparison between other-report and self-report questionnaires would add evidence about rater type in measuring this kind of behavior [39,40].

## Conclusion

This study replicated the original SRS-A study in a Japanese population and extended previous studies on the child version of the SRS to an adult population. That is, the SRS-A distributed continuously in the non-clinical population, and the other-report SRS-A rated by parents, spouses, siblings, and close friends was found to be reliable across gender and age, except in the youngest men aged 19 to 24 years. Furthermore, the SRS-A is useful for detecting ASD and autistic traits/symptoms among psychiatric patients and also for capably discriminating ASD from non-ASD psychiatric disorders such as major depression and schizophrenia. We have recommended optimal cutoff scores feasible for use in clinical settings.

## Abbreviations

ADOS: Autism Diagnostic Observation Schedule
ANOVA: Analysis of variance
AQ: Autism-spectrum quotient
AQ-J: Japanese version of the autism-spectrum quotient
ASD: Autism spectrum disorder
DSM-IV-TR: Diagnostic and Statistical Manual of Mental Disorders, Fourth Edition, Text Revision
DSM-5: Diagnostic and Statistical Manual of Mental Disorders, Fifth Edition
IQ: Intelligence quotient
PARS: Pervasive Developmental Disorders-Autism Society Japan Rating Scale
PDD-NOS: Pervasive developmental disorder-not otherwise specified
SRS-A: Social Responsiveness Scale-Adult version

## References

[1] Brugha TS, McManus S, Bankart J, Scott F, Purdon S, Smith J, Bebbington P, Jenkins R, Meltzer H (2011). Epidemiology of autism spectrum disorders in adults in the community in England. Arch Gen Psychiatry.

[2] Fombonne E (2009). Epidemiology of pervasive developmental disorders. Pediatr Res.

[3] Kamio Y, Inada N, Koyama T (2013). A nationwide survey on quality of life and associated factors of adults with high-functioning autism spectrum disorders. Autism.

[4] Kamio Y, Inokuchi E (2009). Hattasushogaisya to Seishinkairyo no Yakuwari: Saikin no Keiko to Kongo no Kadai [Psychiatric practice’s role for individual with developmental disorders: current trend and future issues]. J Jpn Assoc Psychiatr Hosp.

[5] Cochran DM, Dvir Y, Frazier JA (2013). “Autism-plus” spectrum disorders: intersection with psychosis and the schizophrenia spectrum. Child Adolesc Psychiatr Clin N Am.

[6] American Psychiatric Association (2013). Diagnostic and statistical manual of mental disorders.

[7] American Psychiatric Association (2000). Diagnostic and Statistical Manual of Mental Disorders. Fourth Edition. Text Revision.

[8] Constantino JN, Todd RD (2003). Autistic traits in the general population: a twin study. Arch Gen Psychiatry.

[9] Kamio Y, Inada N, Moriwaki A, Kuroda M, Koyama T, Tsujii H, Kawakubo Y, Kuwabara H, Tsuchiya KJ, Uno Y, Constantino JN (2013). Quantitative autistic traits ascertained in a national survey of 22,529 Japanese schoolchildren. Acta Psychiatr Scand.

[10] Baron-Cohen S, Wheelwright S, Skinner R, Martin J, Clubley E (2001). The autism-spectrum quotient (AQ): evidence from Asperger syndrome/high-functioning autism, males and females, scientists and mathematicians. J Autism Dev Disord.

[11] Kurita H, Koyama T, Osada H (2005). Autism-spectrum quotient-Japanese version and its short forms for screening normally intelligent persons with pervasive developmental disorders. Psychiatry Clin Neurosci.

[12] Wakabayashi A, Baron-Cohen S, Wheelwright S, Tojo Y (2006). The autism-spectrum quotient (AQ) in Japan: a cross-cultural comparison. J Autism Dev Disord.

[13] Ritvo RA, Ritvo ER, Guthrie D, Yuwiler A, Ritvo MJ, Weisbender L (2008). A scale to assess the diagnosis of autism and Asperger’s disorder in adults (RAADS): a pilot study. J Autism Dev Disord.

[14] Ritvo RA, Ritvo ER, Guthrie D, Ritvo MJ, Hufnagel DH, McMahon W, Tonge B, Mataix-Cols D, Jassi A, Attwood T, Eloff J (2011). The Ritvo Autism Asperger Diagnostic Scale-Revised (RAADS-R): a scale to assist the diagnosis of Autism spectrum disorder in adults: an international validation study. J Autism Dev Disord.

[15] Constantino JN, Gruber CP (2012). Social Responsiveness Scale, Second Edition (SRS-2).

[16] Nylander L, Gillberg C (2001). Screening for autism spectrum disorders in adult psychiatric out-patients: a preliminary report. Acta Psychiatr Scand.

[17] Constantino JN, Gruber CP (2005). Social Responsiveness Scale (SRS).

[18] Constantino JN, Gruber CP, Davis S, Hayes S, Passanante N, Przybeck T (2004). The factor structure of autistic traits. J Child Psychol Psychiatry.

[19] Constantino JN, Hudziak JJ, Todd RD (2003). Deficits in reciprocal social behavior in male twins: evidence for a genetically independent domain of psychopathology. J Am Acad Child Adolesc Psychiatry.

[20] Bölte S, Poustka F, Constantino JN (2008). Assessing autistic traits: cross-cultural validation of the social responsiveness scale (SRS). Autism Res.

[21] Fombonne E, Marcin C, Bruno R, Tinoco CM, Marquez CD (2012). Screening for autism in Mexico. Autism Res.

[22] Wang J, Lee LC, Chen YS, Hsu JW (2012). Assessing autistic traits in a Taiwan preschool population: cross-cultural validation of the Social Responsiveness Scale (SRS). J Autism Dev Disord.

[23] Reiersen AM, Constantino JN, Volk HE, Todd RD (2007). Autistic traits in a population-based ADHD twin sample. J Child Psychol Psychiatry.

[24] Noriuchi M, Kikuchi Y, Yoshiura T, Kira R, Shigeto H, Hara T, Tobimatsu S, Kamio Y (2010). Altered whilte matter fractional anisotropy and social impairment in children with autism spectrum disorder. Brain Res.

[25] Duvall JA, Lu A, Cantor RM, Todd RD, Constantino JN, Geschwind DH (2007). A quantitative trait locus analysis of social responsiveness in multiple autism families. Am J Psychiatry.

[26] Marrus N, Faughn C, Shuman J, Petersen S, Constantino J, Povinelli D, Pruett JR (2011). Initial description of a quantitative, cross-species (chimpanzee-human) social responsiveness measure. J Am Acad Child Adolesc Psychiatry.

[27] Constantino JN, Todd RD (2005). Intergenerational transmission of subthreshold autistic traits in the general population. Biol Psychiatry.

[28] Bölte S (2012). Brief report: the social responsiveness scale for adults (SRS-A): initial results in a German cohort. J Autism Dev Disord.

[29] Kuroda M, Inada N, Yukihiro R, Uchiyama T, Hirose K, Uno U, Kamio Y, Uchiyama T (2013). Autism Diagnosis Observation Schedule (ADOS-G): Nihongoban zen module no shinraisei to datousei ni kansuru kenkyu. [Reliability and validity of the Japanese version of ADOS-G, module 1–4]. Annual Report of Research Supported by Health and Labour Sciences Research Grants.

[30] Ito H, Tani I, Yukihiro R, Adachi J, Hara K, Ogasawara M, Inoue M, Kamio Y, Nakamura K, Uchiyama T, Ichikawa H, Sugiyama T, Hagiwara T, Tsujii M (2012). Validation of an interview-based rating scale developed in Japan for pervasive developmental disorders. Res Autism Spect Dis.

[31] Kamio Y, Yukihiro R, Adachi J, Ichikawa H, Inoue M, Uchiyama T, Kurita H, Sugiyama T, Tsujii M (2006). Reliability and validity of the pervasive developmental disorder (PDD)–autism society Japan rating scale (PARS): a behavior checklist for adolescent and adults with PDDs. Clin Psychiatr (Seishin-Igaku).

[32] Kamio Y, Tsujii H, Inada N, Inokuchi E, Kuroda M, Koyama T, Uno Y, Okudera T, Ichikawa H, Takaki A (2009). Validation of the Japanese verion of the social responsiveness scale: comparison with PDD–autism society Japan rating scales (PARS). Clin Psychiatr (Seishin-Igaku).

[33] Frazier TW, Ratliff KR, Gruber C, Zhang Y, Law PA, Constantino JN (2014). Confirmatory factor analytic structure and measurement invariance of quantitative autistic traits measured by the social responsiveness scale-2. Autism.

[34] De Lacy N, King BH (2013). Revisiting the relationship between autism and schizophrenia: toward an integrated neurobiology. Annu Rev Clin Psychol.

[35] Chung YS, Barch D, Strube M (2014). A meta-analysis of mentalizing impairment in adults with schizophrenia and autism spectrum disorder. Schizophr Bull.

[36] Lundstöm S, Chang Z, Kerekes N, Gumpert CH, Råstam M, Gillberg C, Lichtenstein P, Anckarsäter H (2011). Autistic-like traits and their association with mental health problems in two nationwide twin cohorts of children and adults. Psychol Med.

[37] Moriwaki A, Kamio Y (2013). Associations between autistic traits and psychiatric issues in Japanese school children and adolescents. Jap J Autistic Spectrum.

[38] Hofvander B, Delorme R, Chaste P, Nydén A, Wentz E, Ståhlberg O, Herbrecht E, Stopin A, Anckarsäter H, Gillberg C, Råstam M, Leboyer M (2009). Psychiatric and psychosocial problem in adults with normal intelligence autism spectrum disorders. BMC Psychiatry.

[39] Kanne SM, Abbacchi AM, Constantino JN (2009). Multi-informant ratings of psychiatric symptom severity in children with autism spectrum disorders: the importance of environmental context. J Autism Dev Disord.

[40] Ingersoll B, Hopwood CJ, Wainer A, Brent Donnellan M (2011). A comparison of three self-report measures of the broader autism phenotype in a non-clinical sample. J Autism Dev Disord.

